# Evaluating the use of fibrin glue for sealing low-output enterocutaneous fistulas: study protocol for a randomized controlled trial

**DOI:** 10.1186/s13063-015-0966-9

**Published:** 2015-10-07

**Authors:** Xiuwen Wu, Jianan Ren, Gefei Wang, Jianzhong Wang, Feng Wang, Yueping Fan, Yuanxin Li, Gang Han, Yanbing Zhou, Xiaofei Song, Bin Quan, Min Yao, Jieshou Li

**Affiliations:** Department of Surgery, Jinling Hospital, Medical School of Nanjing University, 305 East Zhongshan Road, Nanjing, 210002 China; Department of General Surgery, The 1st Affiliated Hospital of Gannan Medical College, Ganzhou, China; Department of General Surgery, The Affiliated Hospital of Neimenggu Medical University, Huhehaote, China; Department of General Surgery, Chinese Medical University Aviation Hospital, Beijing, China; Department of General Surgery, The 309th Hospital of The Chinese People’s Liberation Army, Beijing, China; Department of Surgery, The Second Hospital of Jilin University, Changchun, China; Department of General Surgery, Affiliated Hospital of Medical College Qingdao University, Qingdao, China; Department of General Surgery, Henan Province People’s Hospital, Zhengzhou, China; Department of Gastrointestinal Surgery, Xuzhou Central Hospital, Xuzhou, China; Department of Surgery, VA Medical Center, 830 Chalkstone Avenue, Providence, USA

**Keywords:** enterocutaneous fistulas, fibrin glue, glue sealing

## Abstract

**Background:**

The management of an enterocutaneous fistula poses a significant challenge to surgeons and is often associated with a costly hospital stay and long-term discomfort. The use of fibrin glue in the fistula tract has been shown to promote closure of low output enterocutaneous fistulas. Our previous nonrandomized study demonstrated that autologous platelet-rich fibrin glue treatment significantly decreased time to fistula closure and promoted closure rates. However, there are several limitations in the study, which may lead to bias in our conclusion. Thus, a multicenter, randomized, controlled clinical trial is required.

**Methods/Design:**

The study is designed as a randomized, open-label, three-arm, multicenter study in nine Chinese academic hospitals for evaluating the efficacy and safety of fibrin glue for sealing low-output fistulas. An established number of 171 fistula patients will undergo prospective random assignment to autologous fibrin glue, commercial porcine fibrin sealants or drainage cessation (1:1:1). The primary endpoint is fistula closure time (defined as the interval between the day of enrollment and day of fistula closure) during the 14-day treatment period.

**Discussion:**

To our knowledge, this is the first study to evaluate the safety and efficacy of both autologous and commercial fibrin glue sealing for patients with low-output volume fistulas.

**Trial registration:**

NCT01828892. Registration date: April 2013.

**Electronic supplementary material:**

The online version of this article (doi:10.1186/s13063-015-0966-9) contains supplementary material, which is available to authorized users.

## Background

An enterocutaneous fistula (ECF) still remains as one of the most complex and challenging complications encountered in surgical practice [1]. Its management often requires a costly hospital stay and multidisciplinary care due to high morbidity and mortality induced by sepsis, hemorrhage, malnutrition and electrolyte disturbances [2, 3]. Staged fistula treatment involves recognition and stabilization, anatomic definitioxn and decision, and definitive operation. With sufficient time, conservative treatment can result in spontaneous fistula closure, with rates varying from 15 % to 71 % [4]. However, this also results in long-term discomfort.

Use of fibrin glue (FG) in the fistula tract has been shown to promote closure of low-output ECFs. As a noninvasive option, it could avoid the risk of developing further dehiscence compared to surgical operations. The application of glue has been reported in recent years for intestinal fistula in case reports [5, 6] and small series [7–10]. Commercial fibrin sealants are usually made from porcine/bovine blood products, whereas autologous adhesives have potential advantages, such as a low risk of infection transmission [11] and provision of a matrix for tissue regeneration [12]. Both are used in clinical practice, but they have not yet been compared to fistula sealing.

Our previous study demonstrated that autologous platelet-rich fibrin glue (PRFG) treatment significantly decreased time the time to fistula closure and promoted closure rates. During the follow-up, no treatment-related adverse events or severe adverse events were observed [13]. The previous study was a prospective, nonrandomized single-center study. There were several limitations in the nonrandomized study, which may have led to bias in our conclusion. Thus, a multicenter, randomized, controlled clinical trial is designed to evaluate the glue application in the treatment of patients with low-output volume ECFs.

### Objectives

The objective of this study was to investigate the efficacy and safety of glue sealing (using PRFG or the commercially available fibrin sealant Yueling™) in the management of patients with low-output ECFs.

### Trial design

The trial is designed as a multicenter, open-label, randomized, controlled trial with three parallel groups (two treatment groups and one control group) and a primary endpoint of fistula closure time during the 14-day treatment period. The randomization will be performed by Internet-based software with a 1:1:1 allocation. No blinding will take place in this study.

## Methods/Design

### Participants, interventions and outcomes

#### Study setting

This is a randomized, open-label, therapeutic trial to investigate glue-sealing therapy, and the trial is being conducted in nine Chinese academic hospitals. The nine study sites are distributed in Jiangsu Province, Jiangxi Province, Shandong Province, Beijing, Henan Province, Neimenggu Region and Jilin Province, which cover most parts of East, North, Northeast and Northwest China. The trial began in March 2014 and is scheduled to be completed by the end of 2017.

#### Eligibility criteria

For inclusion, patients must meet the following criteria:

1. Age ≥18 years.
2. Single tubular ECF.
3. Fistula tract length >2 cm.
4. Low output volume (<200 ml/24 h).
5. Written informed consent.

Subjects presenting with any of the indicated general or indication-specific exclusion criteria will not be included in the trial.

The general exclusion criteria include the following:

1. Subject lacks legal capacity.
2. Subject unable to understand the nature, scope, significance and consequences of this clinical trial.
3. Simultaneously participating in another clinical trial or participated in any clinical trial involving administration of acupuncture within 30 days prior to inclusion.
4. Subject exhibits a physical or psychiatric condition, which at the investigator’s discretion may put the subject at risk, may confound the trial results or may interfere with the subject’s participation in this clinical trial.
5. Known or persistent abuse of medication, drugs or alcohol.
6. Current or planned pregnancy or is nursing.

Indication-specific exclusion criteria include the following:

1. Cancer-infiltrated fistula.
2. Intra-abdominal abscess near the fistulas.
3. Foreign bodies inside the fistula tracts.
4. Distal bowel obstruction.
5. Inflammatory bowel disease.

#### Interventions

Qualified subjects will randomly be assigned to one of three study groups: two treatment groups offered autologous PRFG (Group A) or commercial porcine fibrin sealants (Group B) and one control group with drainage cessation (Group C).

All groups receive standard of care (SOC) before assessment for inclusion. The SOC comprises supportive measures to stabilize the fistula patient, including fluid/electrolyte balance, bowel rest, nutritional replacement, wound care, and antibacterial therapy in patients with signs of systemic sepsis or inflammation with pain. Nasogastric or nasointestinal feeding will be provided whenever possible; otherwise, parenteral nutrition along with 0.9 g/24 h somatostatin (Stilamin, Serono, Switzerland) will be infused intravenously. To assist in remission of infection, normal saline will be dripped continuously to rinse the fistula tracts; at the same time, percutaneous suction drainage of these tracts is conducted. Nursing staff monitor the daily external output by collecting output volumes. Once the daily fistula volumes decrease to 200 ml, the patients will be evaluated by the coordinating center.

For patients in Group A, autologous PRFG is prepared as described previously [14]. The platelet-rich cryoprecipitate and thrombin are obtained from the whole blood of patients in Group A at least 24 h prior to the start point. Blood collecting packs, prepared with anticoagulant (citrate phosphate dextrose), are used for the collection of whole blood (300 to 400 ml). The platelet-rich plasma (PRP) is separated from the blood by centrifugation at 6 min at 1,000 x*g* twice at ambient temperature. The PRP is transferred to a new pack, and the amount will be determined by weight. From 40 g PRP, equally transferred to two new 300 ml empty packs, the euglobulin fraction is precipitated by dilution with 180 ml citric acid (2.84 mM) in each pack. After thorough mixing, the pack is centrifuged at 3,000 x*g* for 5 min at 4 °C. The supernatant is removed and the precipitate dissolves in 1.625 ml CaCl_2_ (0.1 M). The pH is adjusted by the addition of 1.0 ml NaHCO_3_ (75 mM) in order to initiate the activation of the prothrombin to thrombin [15]. The clot formation is terminated after 30 minutes, and the liquid thrombin solution is removed into microcentrifuge tubes. At the same time, the cryoprecipitate is produced from the rest of the plasma, which is frozen at −80 °C for at least 6 h first and then thawed at 4 °C. After centrifugation at 4,000 rpm/min for 8 min, the cryoprecipitate is harvested by removal of the cryoprecipitate-poor plasma. When applied, fibrinogen and thrombin are thawed at 37 °C from −20 °C until recovery of the liquid state.

Patients in Group B receive glue sealing with commercial sealants. The Porcine Fibrin Sealant Kit (Yueling®, Hangzhou Puji Medical Technology Co. Ltd, Hangzhou, China) is composed of freeze-dried powder of fibrinogen and thrombin and of relevant solutions. Stored at 4 °C, the kit needs to reach room temperature. Then fibrinogen (Component I, > 30 mg/ml) is dissolved by sodium chloride solution (Activator I, 6.0 to 7.0 mg/ml), and thrombin (Component II, 450 to 850 IU/ml) is dissolved by calcium chloride solution (Activator II, 35.0 to 45.0 mg/ml).

When receiving glue sealing, the patients in the two treatment groups will have drainage cessation. Two main components of FG are placed separately in a double-syringe system with distal mixing catheter. When applied, equal amounts of fibrinogen and thrombin are mixed and the fibrin sealants are to form within 10 seconds. In order to assure total occlusion of the internal hole, the full length of the fistula tract must be determined by image examination prior to the procedure or with assistance of endoscopy [16].

The glue-sealing procedure could be repeated up to three times within the 14-day glue treatment period. A repeat sealing could be conducted if the fistula persists for 5 days after the first procedure. After the 14-day period, patients who fail to achieve medical closure are evaluated to further exclude possible factors disturbing healing, such as infection, foreign body, *etcetera.* These unhealed fistulas will either be treated by definitive surgery or continue to be treated conservatively based on the patient’s best interest and the physicians’ discretion.

Once glue sealing is applied, parenteral nutrition that provides calories of 20 to 25 kcal/kg is initiated through the peripheral vein or central venous catheter. Fistula drainage will be evaluated every day until the drainage decreases to zero, which is defined as fistula closure [17]. It also needs confirmation by contrast enhanced computed tomography at each follow-up visit to verify the complete closure of internal and external openings of the fistula tract. When the closure is achieved, a nasointestinal tube is to be placed and the enteral feeding (2,000 ml/d) is restarted gradually. Other SOC measures can be conducted when needed.

#### Outcomes

The primary outcome of the trial is fistula closure time (defined as the interval between the day of enrollment and day of fistula closure) during the 14-day treatment period. Fistula closure is defined as complete closure of the fistula tract and internal and external openings later, which will be confirmed by contrast enhanced computed tomography at each follow-up visit where there is no drainage or any sign of inflammation [17].

In addition to the primary outcome measure of fistula closure, secondary outcomes include closure rates up to 14 days, closure rates up to 180 days, fistula recurrence rates and the incidence of adverse events and severe adverse events up to 180 days (defined as an event that was fatal or life-threatening, led to additional hospitalization or disability or required an intervention to prevent one of these outcomes).

#### Participant timeline

**Fig. 1:**
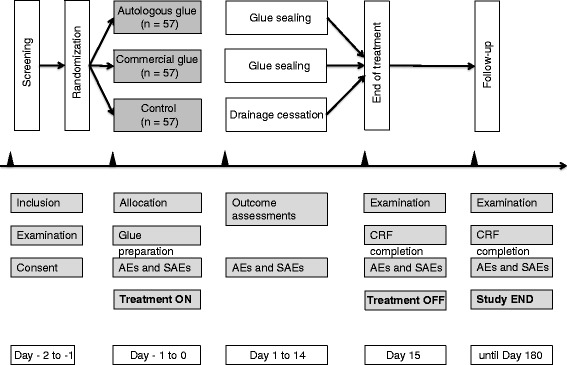
Study design. Patients are randomly allocated to group A (autologous fibrin glue), group B (commercial fibrin glue) or group C (drainage cessation). After glue application or drainage cessation, fistula drainage will be evaluated for 14 days, during which a maximum of three times of glue sealing will be conducted for patients in groups A and B. Both efficacy and safety outcomes will be assessed during the whole study period.

All patients with ECFs in each study site receive SOC, and the fistula output will be evaluated every day. In case of daily output less than 200 ml, the inclusion criteria will be assessed, and imaging examination will be performed to exclude abscesses, obstruction, *etcetera.* Then participants are randomly allocated to Group A (autologous fibrin glue), Group B (commercial fibrin glue) or Group C (drainage cessation). Once enrolled and allocated to Group A, autologous PRFG needs to be prepared. The study period is set as 14 days after the day of enrollment, and fistula drainage will be evaluated every day. All patients are followed up for at least 180 days. The outpatient visits will be scheduled for 1 month, 3 months and 6 months after enrollment (Fig. 1).

#### Sample size

The sample size is to be calculated based on detecting the minimal, clinically relevant difference in the success rates of the three arms. Our preliminary study, which enrolled 145 patients, compared the efficacy of PRFG application (PRFG group) with drainage cessation (control group) when low output volume was achieved (<200 ml/d). During the 28-day treatment period, 77 % (58/75) of the patients in the PRFG group experienced fistula closure in comparison to 57 % (40/70) in the control group (*P* = 0.0127) [13]. The unpublished data of the pilot study demonstrated that closure rates were 80 % (60/75) in the PRFG group and 50 % (35/70) in the control group during the 14-day observation period. Due to the above raw data, the closure rates in the two treatment groups and one control group are assumed to be 80 % and 50 %, respectively. In this way, 51 patients per group are needed for this trial based on an alpha of 0.05, with a power of 90 %. Taking into account a drop-out rate of 10 %, a total sample size of 171 patients would be included, with 57 participants in each group.

### Assignment of interventions

#### Coordination

A clinical coordinating center in each participating site is established to identify subject eligibility for the study and to provide support to investigators and study personnel throughout the study period. The coordinating center consists of a principle investigator and a well-trained study coordinator. Research coordinators are required to screen all fistula patients in each study center for eligibility once a day, either the morning or afternoon. The patient cannot be enrolled until their eligibility is confirmed by the treating physician. Then, the study coordinator will obtain written informed consent from the patient. Patients will be consecutively enrolled at each of the participating centers whenever a study coordinator is available for enrollment. Consent will be obtained from participating clinicians before or at the time of patient consent.

#### Allocation

Subjects approved for randomization because they meet full eligibility criteria will be randomly assigned to one of the following study groups by using the Internet-based randomization software RANDOULETTE™ (Institute of Medical Information Sciences, Biometry and Epidemiology, University of Munich). Stratification for age will be performed, and an equal distribution between treatment arms (ratio of 1:1:1) will be warranted. The principle investigator in each coordinating center informs clinicians of the allocation sequence via a central telephone to ensure concealment.

#### Blinding

No blinding will take place in this study. Eligible patients who have given consent will be randomized into any of the three groups.

### Data collection, management and analysis

#### Data collection and management

A case report form is used to collect the data for each patient. Assessments are made at the screening period, inclusion (D0) and on days 1 to 14. Assessments include fistula assessment (location, output, duration, and length of fistula tract), monitoring (drainage, catheter, tubes, and adverse events), laboratory tests (hematology, biochemistry, urinalysis, and pregnancy test at inclusion only), and microbiology (blood cultures and cultures of isolates from fistula tracts). Data on adverse events, compliance and concomitant treatments are collected at each visit. Participants will be evaluated every day until the 14th day after randomization. Physicians and research nurses who are not involved in the patients’ care will assess the outcome.

All documents collected in this study will be stored safely in confidential conditions. On all study-specific documents, other than the signed consent, the participant will be referred to by the study participant number/code, not by name. Study documentation will be archived for a period of 5 years after the study.

#### Statistical analysis

The intention-to-treat approach is utilized to examine differences in the fistula closure time among the three groups in the primary analysis by the Pearson χ2 test. Safety parameters and secondary efficacy parameters will be analyzed using the Pearson χ2 test and logistic regression (if necessary). Cox proportional hazards models and the Kaplan-Meier method will also be applied. Adverse events will be tabulated according to randomized group assignment and the proportions will be compared using Fisher’s exact test.

### Monitoring

#### Data monitoring

A Data Monitoring Committee (DMC) has been established. The DMC is independent of the study organizers. During the period of recruitment to the study, interim analyses will be supplied together with any other analyses that the committee may request. This may include analyses of data from other comparable trials. The frequency of interim analyses will depend on the judgement of the Chair of the DMC (MY), in consultation with the study organizers. However, we anticipate that there might be two interim analyses and one final analysis. An interim analysis is performed on the primary endpoint when 50 % of the patients have been randomized and have completed the 6-month follow-up. The trial will not be stopped in case of futility.

#### Harms

All adverse events will be recorded and closely monitored until resolution or stabilization or until it has been shown that the study intervention is not the cause of the event. The chief investigator will be informed immediately of any serious adverse events and will determine (in cooperation with the treating physicians) the seriousness and causality of these events.

All treatment-related serious adverse events will be recorded and reported to the Ethics Committee as part of the report. Unexpected serious adverse events will be reported to the Ethics Committee within the relevant time frames. The chief investigator will be responsible for all adverse event reporting. All site staff will be appropriately trained in the procedures to follow and the forms to use during the study protocol prior to study initiation. Regular central monitoring for all studies and site monitoring, as determined by the trial-specific risk assessment, will be used to ensure that all adverse events are identified and acted on appropriately.

### Ethics and dissemination

#### Ethics committee and regulatory approval

The research team ensures that this study is being conducted in accordance with the principles of the Declaration of Helsinki [18]. The trial will also be conducted in accordance with both international and Chinese ethical guidelines for biomedical research involving human subjects [19, 20].

The study protocol, informed consent form, participant information sheet, and any further patient documents have been submitted to the Institutional Review Board of the Ethics Committee of each study site for written approval. Details of all approving institutional review boards can be found in Additional file 1. Approval has been obtained from the Ethics Committee for all substantial amendments to the documents originally approved.

#### Protocol amendments

Any modifications to the protocol, including changes of study objectives, study design, patient population, sample size, study procedures or significant administrative aspects, may have impacts on the implementation of the trial, potential benefits of patients and the patients’ safety. The modifications will require a formal amendment to the protocol. Such amendments will be agreed on by the sponsor and approved by the Ethics Committee prior to implementation, and the health authorities will be notified in accordance with local regulations.

Administrative changes to the protocol are minor corrections that have no effect on the way the study is to be conducted. These changes will be agreed upon by the sponsor and will be documented in a memorandum. The Ethics Committee will be notified of administrative changes.

## Discussion

To our knowledge, this is the first study to evaluate the safety and efficacy of both autologous and commercial fibrin glue sealing for patients with low-output fistulas. Currently, fibrin glue is indicated only in treating simple ECFs of low-output volume as a first line therapeutic option. Therefore, complex or high-output ECFs are excluded from this study. In addition, this study is designed as an open-label, three-arm study, with autologous and commercial fibrin glue in two treatment groups and drainage cessation for use as a placebo. Because the autologous fibrin sealants require blood donation from patients themselves, patient blinding cannot be conducted. Further, there is a possibility that spontaneous closure can be achieved for fistula patients. Therefore, the control group is being established for comparison of the efficacy and safety of the glue application. In this way, this study is designed as an open-label and three-arm study.

## Trial status

The study was conceived and designed in 2014. We are now recruiting participants.

## Additional file

Additional file 1:
**Ethical bodies approved the study.** Details for the approving institutional review boards. (DOCX 67 kb)

## Abbreviations

ECF: enterocutaneous fistula
FG: fibrin glue
PRFG: autologous platelet-rich fibrin glue
PRP: platelet-rich plasma
SOC: standard of care
