# Supplementary material for: Evaluating the use of fibrin glue for sealing low-output enterocutaneous fistulas: study protocol for a randomized controlled trial
Source: Trials. 2015 Oct 7;16:445. doi: 10.1186/s13063-015-0966-9 (PMC4597766; doi:10.1186/s13063-015-0966-9)
Supplement: Additional file 1: — Ethical bodies approved the study. Details for the approving institutional review boards. (DOCX 67 kb) [file 13063_2015_966_MOESM1_ESM.docx]

**Additional file**

| **Table1 Ethical bodies approved the study** | | | |
| --- | --- | --- | --- |
| **Site #** | **Institution** | **IRB address** | **Approval date** |
| Site #1 | The 1st Affiliated Hospital of Gannan Medical College | 23 Qingnian Rd, Ganzhou 341000, Jiangxi Province, China | 12 Mar 2014 |
| Site #2 | Henan Province People's Hospital | 7 Weiwu Rd, Jinshui District, Zhengzhou 450000, Henan Province, China | 5 Mar 2014 |
| Site #3 | The Second Hospital of Jilin University | 18 Ziqiang St, Nanguan District, Changchun 130000, Jilin Province, China | 17 Mar 2014 |
| Site #4 | The 309th Hospital of The Chinese People`s Liberation Army | 17 Heishanfulujia Rd, Haidian District, Beijing 100091, China | 3 Feb 2014 |
| Site #5 | Jinling Hospital | 305 East Zhongshan Rd, Xuanwu District, Nanjing 210002, China | 20 Mar 2014 |
| Site #6 | The Affiliated Hospital of Neimenggu Medical University | 1 Xinhuada St, Huhehaote 010110, Neimenggu, China | 7 Jan 2014 |
| Site #7 | Affiliated Hospital of Medical College Qingdao University | 16 Jiangsu Rd, Qingdao 266071, Shandong Province, China | 26 Mar 2014 |
| Site #8 | Xuzhou Central Hospital | 199 South Jiefang Rd, Quanshan District, Xuzhou 221008, Jiangsu Province, China | 14 Mar 2014 |
| Site #9 | Chinese Medical University Aviation Hospital | 3 Anwaibeiyuan, Chaoyang District, Beijing 10000, China | 31 Jan 2014 |
| IRB, Institutional Review Board  Sites were sorted according to the initial of Chinese phonetic alphabet. | | | |
